# Fistula occlusion and ligation for a giant right coronary artery aneurysm concurrent with right atrial fistula: a case report

**DOI:** 10.1186/s12893-019-0624-3

**Published:** 2019-11-08

**Authors:** Yan Ren, Lin Xie, Weiqiang Ruan, Yajiao Li, Peng Ji, Changping Gan, Ke Lin

**Affiliations:** 1 Department of Cardiovascular Surgery, West China Hospital, Sichuan University, No. 37 Guoxue Lane, Wuhou District, Chengdu, 610041 Sichuan China; 2 Department of Cardiology, West China Hospital, Sichuan University, Chengdu, Sichuan China; 3 Department of Critical Care Medicine, West China Hospital, Sichuan University, Chengdu, Sichuan China

**Keywords:** Coronary artery aneurysm, Coronary artery fistula, Cardiopulmonary bypass

## Abstract

**Background:**

Coronary artery aneurysms in most cases require surgical treatment once diagnosed. Lifelong anticoagulation is often needed after surgery. We herein describe a 55-year-old man who was asymptomatic and diagnosed with right giant coronary artery aneurysm combined with right atrial fistula.

**Case presentation:**

This is a case of asymptomatic giant right coronary artery aneurysm concurrent with coronary artery fistula. Because the aneurysm was in the distal right posterior descending coronary artery, right coronary artery ligation and fistula occlusion through the right atrium were performed in the absence of cardiopulmonary bypass. The aneurysm was excluded without impacting the myocardial blood supply, and the patient was exempted from lifelong anticoagulation regimen. The follow-up revealed favorable outcomes and the patient’s life expectancy was improved.

**Conclusion:**

Decompression and exclusion without cardiopulmonary bypass can be adopted for distal coronary artery aneurysms that do not involve or only have a limited impact on distal blood supply. This procedure can exempt the patient from the lifelong anticoagulation regimen. In addition, the risk for myocardial ischemia caused by the thrombus in the aneurysm can also be avoided. The whole procedure is comparatively easy to perform.

## Background

Coronary artery fistula (CAF) is a rare congenital anatomical anomaly with an incidence of about 0.2% [1, 2]. CAF is associated with aneurysms in 19% of the cases. Moreover, these aneurysms may fistulate to the right atrium with an incidence of 26% [3]. An aneurysm > 20 mm in diameter is termed as a giant coronary aneurysm [4]. Though the underlying mechanism remains unknown, it is believed that the increased blood flow and pressure destroy the media structure of the vascular wall and decrease the elasticity of the coronary artery, thereafter arousing the concurrent aneurysm. In adults, atherosclerosis is the most common cause for coronary artery aneurysm (CAA), which accounts for 50% of all the cases [5, 6]. CAA of all sizes may rupture and the risk of rupture increases in a size-dependent manner [7]. Prompt surgery is necessary for patients with evident symptoms of CAA. Yet for asymptomatic patients, whether surgery is a necessity is controversial. Surgical treatment is recommended to asymptomatic patients due to the low natural closure rate of fistula and the potential complications of aneurysm like myocardial ischemia, bacterial endocarditis and coronary artery aneurysm. This paper reports a case of asymptomatic a giant right coronary artery aneurysm concurrent with coronary artery fistula. Surgery was performed in the absence of cardiopulmonary bypass (CPB) and the patient was exempted from a lifelong postoperative anticoagulation regimen.

## Case presentation

**Fig. 1:**
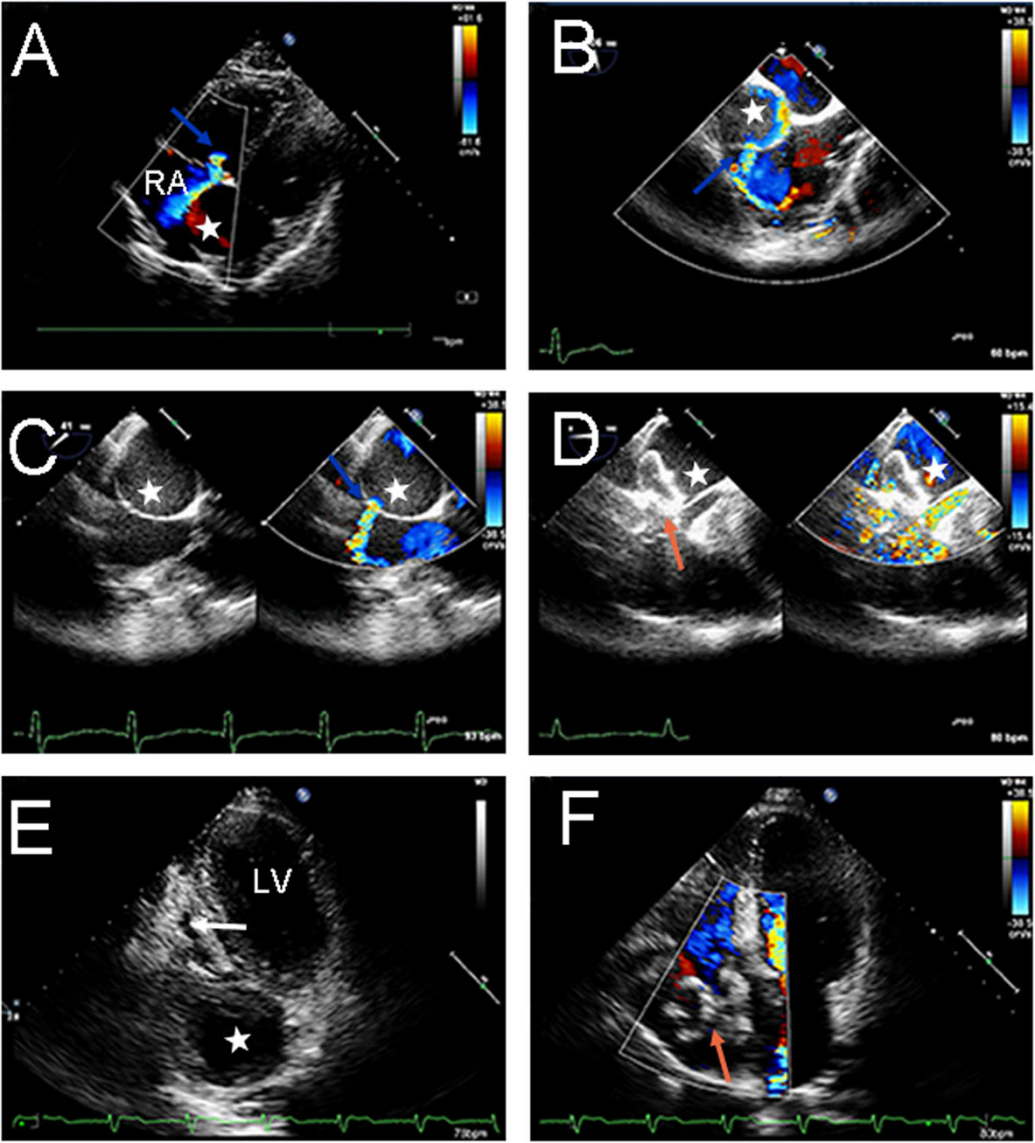
Echocardiogram before, during and after 3 months of operation findings: **a**, **b** Pre-operation echocardiography demonstrated a giant right coronary artery aneurysm (65*48 mm) (white star) with a fistula to the RA (blue arrow); **c**, **d** Intraoperative TEE showed AAU and fistula. A 30 mm ASD occluder (red star) was placed; **e**, **f** 3 months post-operation, TTE revealed a residual aneurysm of about 42*42 mm, mural thrombosis in the aneurysm, and no residual shunt between RCA and RA was found. The visual segment of the right coronary artery was still dilated (white star). (RA right atrium; TTE transthoracic echocardiography; ASD atrial septal defect; RCA right coronary artery; RA right atrium; LV Left ventricle)

**Fig. 2:**
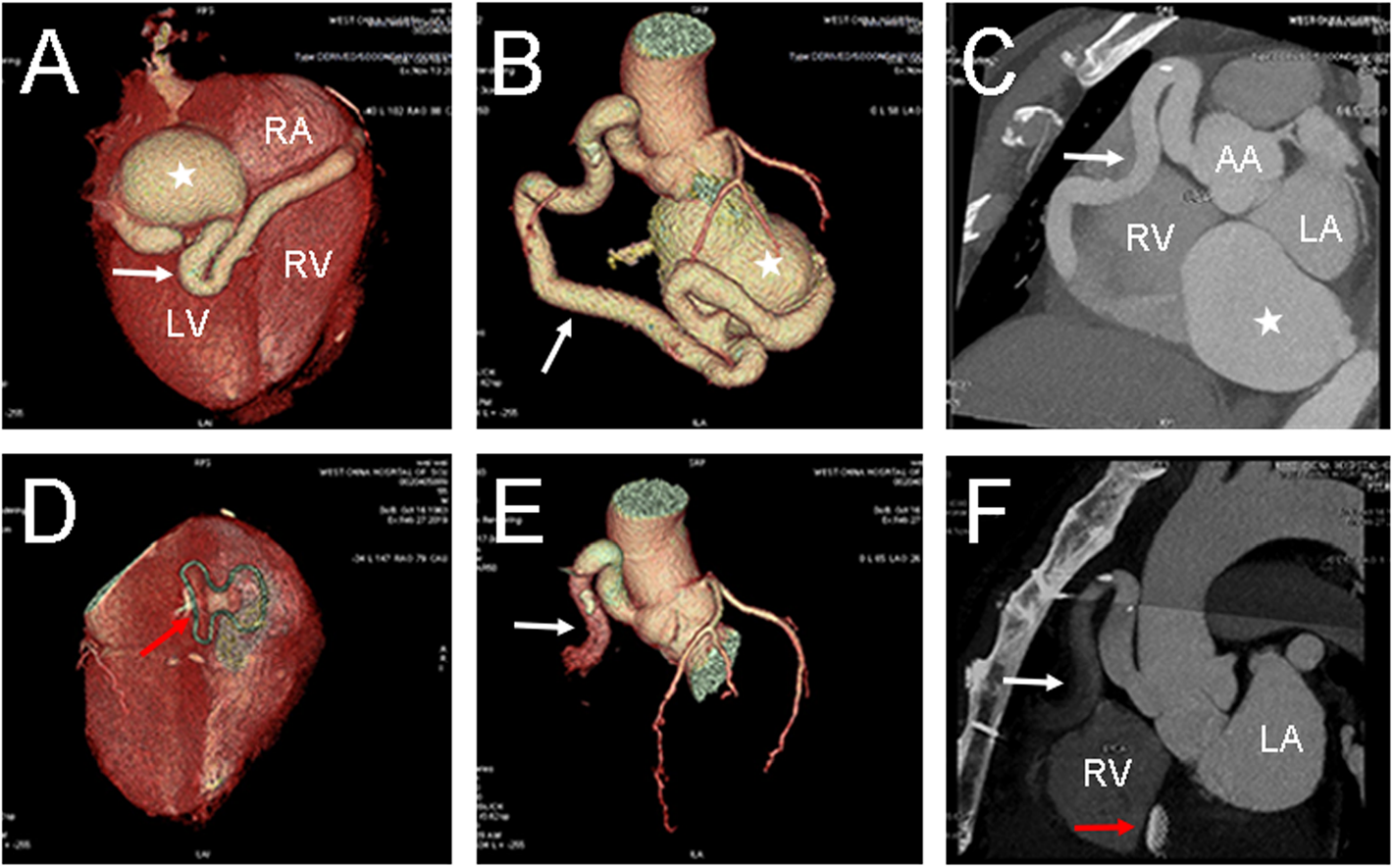
Preoperative and Postoperative CT findings: **a**, **b**, **c** Pre-operation CT showed tortuous and dilated RCA (white arrow) and the diameter increased to 11 mm, a giant aneurysm (65* 48 mm) (white star) originating from the distal RCA and draining into the RA; **d**, **e**, **f** In the third postoperative months, there was no displacement of the occluder (red arrow) and the thrombus formed at the distal end of the right coronary artery was undeveloped.(RCA right coronary artery; LA left atrium; RA right atrium; RV right ventricle; AA ascending aorta)

**Fig. 3:**
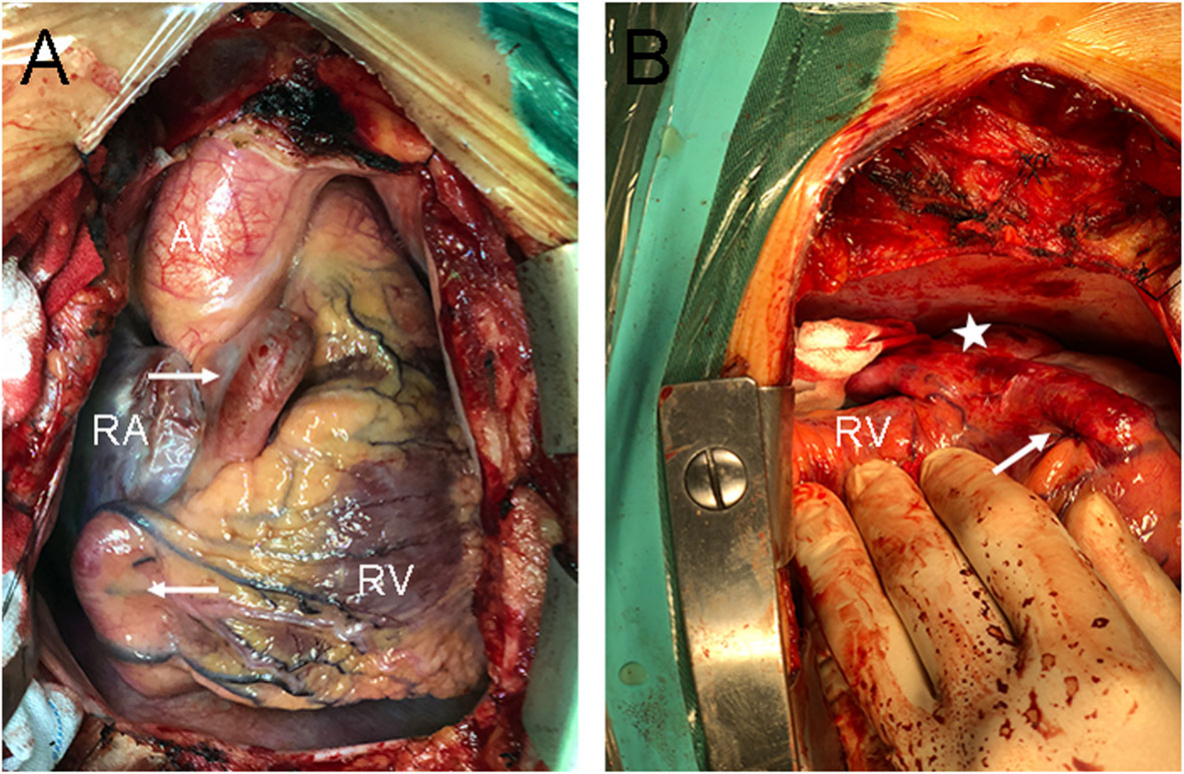
Intraoperative findings: **a** Intraoperative confirmed tortuous and dilated RCA (white arrow); **b** aneurysmal dilation behind the right ventricle (white star). (RCA right coronary artery; RA right atrium, RV right ventricle, AA, ascending aorta)

**Fig. 4:**
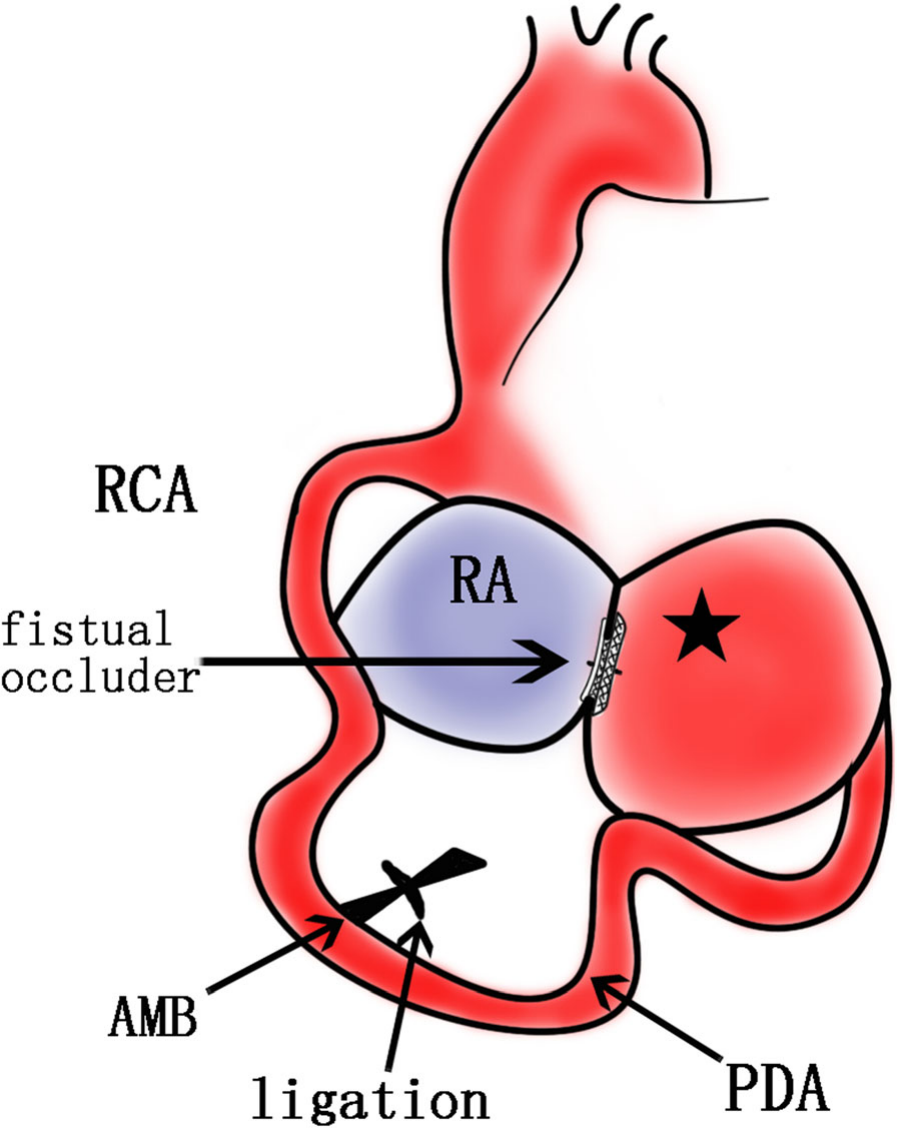
A schematic diagram of the operation: RCA significantly tortuous and dilated, the ligation was performed 3 cm at the distal end of the AMB, and then the fistula was occluded through the RA. (★ CAA, coronary artery aneurysm; RCA right coronary artery, AMB acute marginal branch, RA right atrium)

A 55-year-old man was admitted to the Cardiovascular Surgery Department of our hospital for CAA detected by transthoracic echocardiography (TTE) during a routine physical examination. He had neither symptoms of cardiac diseases such as dyspnea, fatigue, chest distress, angina or arrhythmia nor family history of any cardiac diseases. Physical examination showed his heart rate was 78 beats per minute and the blood pressure was 138/80 mmHg. Grade III to-and-fro murmur was heard over the second intercostal space of his left sternal margin. Electrocardiogram (ECG) showed normal sinus rhythm and no significant ST segment or T wave anomaly was found. Chest radiography showed enlarged heart shadow. TTE demonstrated a giant right coronary artery (RCA) aneurysm with a fistula to the right atrium (RA), and the ejection fraction(EF) was 58% (Fig. 1a, b). The diameter of the RCA increased to 11 mm. Coronary artery computed tomography (CT) revealed a giant aneurysm (65*48 mm) originating from the distal RCA and draining into the RA (Fig. 2a, b, c). The results of coronary angiography (CAG) were consistent with the above results. Considering the giant size of the aneurysm, surgery was needed as early as possible. Median sternotomy revealed tortuous and dilated RCA, as well as aneurysmal dilation behind the right ventricle (Fig. 3a, b); a lateral branch originating from the acute marginal branch (AMB) of the RCA and being connected to the aneurysm. Since the surgery aimed to decompress the aneurysm and eliminate the shunt at the fistula site, identifying the entrance and exit vessels of the aneurysm was of vital necessity. To reduce the blood flow and pressure by closing the entrance vessel as much as possible without causing myocardial ischemia, the proximal RCA was ligated first. But intraoperative ECG monitoring showed elevated ST segment, ischemic myocardium and weakening contraction of the right ventricle (RV). Hence, the ligation was performed 3 cm from the distal end of the AMB. After ligation, no elevated ST segment was found. Transesophageal echocardiography (TEE) showed no ventricular wall motion abnormalities and the aneurysm was obviously downsized. The fistula was then occluded through the RA under TEE guidance in the absence of CPB. First, the guide wire and catheter sheath were inserted and passed through the fistula. Then a 30 mm atrial septal defect (ASD) occluder (BEIJING HUAYISHENGJIE) was applied to achieve maximal closure of the aneurysm (Fig. 4). TEE showed the size of the aneurysm was obviously reduced, and no blood flow signals, ventricular wall motion abnormalities or shunt were observed (Fig. 1c, d). The patient was discharged 9 days after operation. Aspirin and clopidogrel were administered for 3 months. The follow-up revealed favorable outcomes and an improved quality of life for the patient.

## Discussion and conclusion

Although conventional CAA surgery under CPB has some possible complications, including intraoperative ischemia, impaired hemostasis and mechanical trauma to blood cells [8, 9], many surgeons still believe it is the “gold-standard” operation for this type of pathology. In the present case, the surgery was performed in the absence of CPB procedure not only to avoid the CPB- associated complications [10], but also to monitor cardiac ischemia in “real-time” and facilitate repair.

In the present case, the giant CAA was formed at the distal right posterior descending coronary artery (PDA). Since PDA is connected to the left anterior descending coronary artery (LAD) through small branches, the fistula orifice in the aneurysm could “steal” blood from the posterior descending branch of the RCA. So, when myocardial ischemia occurs, the LAD might compensatorily extend and supply blood to this ischemic part. It explained why the patient had no symptoms of myocardial ischemia prior to the surgery. Typically, if the surgical treatment had not been applied, the patient would have been on a long-term antiplatelet therapy to avoid the development of thrombus in the enlarged coronary artery (CA) and CAA. However, long-term medication would inevitably bring negative impacts on the patient’s physical and mental health, which would then impair the patient’s quality of life. Furthermore, even if on medication, he would still face the risks of thrombosis in the aneurysm, myocardial ischemia, rupture of aneurysm or other life-threatening complications. Now since surgery was performed and the size of the aneurysm was reduced, the risks mentioned above have been significantly reduced. Compared with lifelong anticoagulation therapy, the three-month medication lasted for a fairly short period. So, we believe the patient’s life expectancy might be improved by the surgery.

So far, no consensus has been reached on anticoagulant therapy after operation. Warfarin seems workable for patients with CAA [11]. After CAF occlusion, whether anticoagulation is needed and how to perform it is yet to be determined. All the currently available studies regarding this issue have a small sample size. Okubo reported the postoperative use of aspirin in small doses after the application of Amplatzer, the ductus arteriosus occluder [12]. In Collins’ study, most patients were not given anticoagulant [13], except two receiving aspirin or the combination of aspirin and clopidogrel for the slowed blood flow in CA. In the present case, since the patient had dilated and tortuous RCA and slowed blood flow, aspirin and clopidogrel, as antiplatelet agents, were administered for 3 months, as in the case of ASD occlusion. In the postoperative 3-month follow-up, the patient did not complain of chest distress or chest pain. ECG did not indicate myocardial ischemia. Doppler echocardiography revealed a residual aneurysm of about 42*42 mm and mural thrombosis in the aneurysm. The ventricular wall motion was normal, and no residual shunt between RCA and RA was found (Fig. 1e, f). Postoperative LVEF, LA, RA, RV diameter, Tricuspid Annular Plane Systolic Excursion(TAPSE), S′ velocity of Doppler tissue imaging (DTI-S′) were 59%, 34 mm, 41 mm, 30 mm, 13 mm, 8 cm/s, respectively. Compared with the preoperative parameters (58%, 41 mm, 46 mm, 30 mm, 15 mm, 9 cm/s, respectively), all parameters have been reduced except RV diameter. CT of CA showed the fixed occluder, dilated proximal RCA, and unshadowed thrombosis in the distal RCA and residual aneurysm (Fig. 2d, e, f). As shown in Table 1. The cardiac markers and natriuretic peptides soared rapidly on the first postoperative day and then decreased gradually. On the ninth postoperative day, when the patient was discharged, Troponin-T, Myoglobin, CK-MB were all normal except for natriuretic peptide. In the postoperative 3-month follow-up, all the markers turned normal. If the myocardial markers are elevated due to RCA branch ligation, we speculate that the myocardial markers should be maintained at high levels or continue to increase after surgery. Hence, we speculate that the increase of cardiac markers and natriuretic peptides was caused by the impact of surgery itself rather than the occlusion of RCA.

**Table 1:** Comparison of cardiac markers and natriuretic peptides

	Normal range	Pre-operation	Post-operative day 1	Post-operative day 2	Post-operative day 5	Post-operative day 7	Discharge Post-operative day 9	3months post-operation
Natriuretic peptide	0-227pg/ml	91	2522	2137	1296		618	231
Troponin-T	0-14ng/L	6.9	648.7	590.7	954.9	485.1	15.3	8.7
Myoglobin	<72ng/ml	<21	98.99	91.86	22.57	<21	25.51	<21
CK-MB	<4.94ng/ml	0.64	45.08	12.25	0.68	0.58	0.89	0.89

In conclusion, decompression and exclusion can be adopted for distal CAA that does not involve or only has a limited impact on distal blood supply. This procedure can spare the patient the lifelong anticoagulation regimen. In addition, the risk for myocardial ischemia caused by the thrombus in the aneurysm can also be avoided. The whole procedure is comparatively easy and the patient’s quality of life can be remarkably improved. Individualized fistula treatment should be designed based on the location of the fistula and the maximized benefits for the patient.

## Data Availability

The datasets used and analyzed during the current study are available from the corresponding author on reasonable request.

## Abbreviations

AMB: Acute marginal branch
ASD: Atrial septal defect
CA: Coronary artery
CAA: Coronary artery aneurysms
CAG: Coronary angiography
CPB: Cardiopulmonary bypass
CT: Computed tomography
DTI-S′: S′ velocity of Doppler tissue imaging
ECG: Electrocardiogram
EF: Ejection fraction
LA: Left atrium
LAD: Left anterior descending coronary artery
LVEF: Left ventricle ejection fraction
PDA: Posterior descending coronary artery
RA: Right atrium
RCA: Right coronary artery
RV: Right ventricle
TAPSE: Tricuspid Annular Plane Systolic Excursion
TEE: Transesophageal echocardiography
TTE: Transthoracic echocardiography

## References

[1] Kang SM, Kim JH, Oh J, Shim CY, Choi BW (2009). Cardiovascular images. Giant right coronary aneurysm to left ventricular fistula. Circ Cardiovasc Imaging.

[2] Buccheri D, Chirco PR, Geraci S, Caramanno G, Cortese B (2018). Coronary artery fistulae: anatomy, diagnosis and management strategies. Heart Lung Circ.

[3] Zenooz NA, Habibi R, Mammen L, Finn JP, Gilkeson RC (2009). Coronary artery fistulas: CT findings. RadioGraphics.

[4] Tokunaga C, Imai A, Enomoto Y, Tanaka YO, Matsushita S, Hiramatsu Y, Sakakibara Y (2010). Giant coronary artery aneurysm with pulmonary artery fistula in a patient on chronic hemodialysis. Ann Thorac Surg.

[5] Li D, Wu Q, Sun L, Song Y, Wang W, Pan S, Luo G, Liu Y, Qi Z, Tao T (2005). Surgical treatment of giant coronary artery aneurysm. J Thorac Cardiovasc Surg.

[6] Shen J, Zhou Y, Fang Z, Hu J (2019). Multiple giant coronary artery aneurysms combined with right coronary artery-pulmonary artery fistula: a case report. BMC Surg.

[7] Hironori Inoue M, Masahiro Ueno MD (2009). Surgical treatment of coronary artery aneurysm with coronary artery fistula. Ann Thorac Cardiovasc Surg.

[8] Liu JC, Chan P, Chang TH, Chen RF (2006). Off-pump surgery for multiple coronary artery fistulas with aneurysm. Ann Thorac Surg.

[9] Magovern JA, Mack MJ, Landreneau RJ (1996). The minimally invasive approach reduces the morbidity of coronary artery bypass. Circulation.

[10] Akins CW, Boucher CA, Pohost GM (1984). Preservation of interventricular septal function in patients having coronary artery bypass grafts without cardiopulmonary bypass. Am Heart J.

[11] Crawley PD, Mahlow WJ, Huntsinger DR, Afiniwala S, Wortham DC (2014). Giant coronary artery aneurysms: review and update. Tex Heart Inst J.

[12] Okubo M, Nykanen D, Benson LN (2001). Outcomes of Transcatheter Embolization in the Treatment of Coronary Artery Fistulas. Catheter Cardiovasc Interv.

[13] Collins N, Mehta R, Benson L, Horlick E (2007). Percutaneous coronary artery fistula closure in adults: technical and procedural aspects. Catheter Cardiovasc Interv.

